# 40 Hz visual stimulation during sleep evokes neuronal gamma activity in NREM and REM stages

**DOI:** 10.1093/sleep/zsae299

**Published:** 2024-12-19

**Authors:** Laura Hainke, James Dowsett, Manuel Spitschan, Josef Priller

**Affiliations:** Department of Psychiatry and Psychotherapy, TUM School of Medicine and Health, Technical University of Munich, Munich, Germany; Department of Psychology, Ludwig Maximilian University, Munich, Germany; Department of Health and Sport Sciences, TUM School of Medicine and Health, Technical University of Munich, Munich, Germany; Department of Psychology, University of Stirling, Stirling, UK; Department of Health and Sport Sciences, TUM School of Medicine and Health, Technical University of Munich, Munich, Germany; Translational Sensory & Circadian Neuroscience, Max Planck Institute for Biological Cybernetics, Tübingen, Germany; TUM Institute of Advanced Study (TUM-IAS), Technical University of Munich, Garching, Germany; Department of Psychiatry and Psychotherapy, TUM School of Medicine and Health, Technical University of Munich, Munich, Germany; Neuropsychiatry, Charité – Universitätsmedizin Berlin and DZNE, Berlin, Germany; Centre for Clinical Brain Sciences, University of Edinburgh and UK DRI, Edinburgh, UK; German Center for Mental Health (DZPG), Munich, Germany

**Keywords:** electroencephalography, gamma, 40 Hz, flicker, visual stimulation, NREM sleep, REM sleep, steady-state visually evoked potential

## Abstract

**Study Objectives:**

Visual stimulation (VS) at 40 Hz is being tested as a non-invasive approach against dementias such as Alzheimer’s disease. Applying it during sleep could increase the convenience, duration, and efficacy of stimulation. Here, we tested the feasibility of 40 Hz VS during sleep in a proof-of-concept study.

**Methods:**

Thirty healthy participants underwent one control and one experimental night of polysomnography at the sleep laboratory. 40 Hz VS was delivered in wakefulness (W), NREM sleep stages 2 and 3, and REM sleep.

**Results:**

As expected, 40 Hz EEG spectral power was increased in all four stages in the experimental condition, compared to control. It was highest in W and similar across NREM 2, NREM 3, and REM, with large and medium effect sizes, respectively. Steady-state visually evoked potential (SSVEP) analyses in the time domain confirmed the specificity of the effect. Secondary analyses revealed that the intervention did not impair objective and subjective sleep quality beyond the first-night effect.

**Conclusions:**

40 Hz VS during sleep effectively evoked neuronal gamma activity at stimulation frequency without degrading sleep quality, supporting the feasibility of this approach. These findings lay the groundwork for optimizing gamma-band sensory stimulation as a tool to causally study cognitive functions and as a scalable, non-invasive intervention against dementias.

## Graphical Abstract

**Graphical Abstract:**
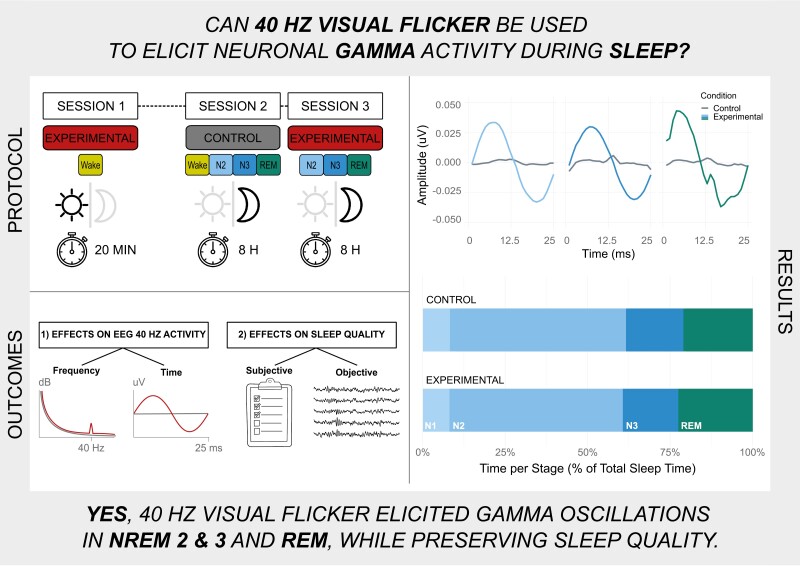

**Statement of Significance**

Neuronal oscillations in the gamma band (especially at 40 Hz) underlie important cognitive processes and can be disrupted in dementias. Visual stimulation (VS) can modulate these oscillations non-invasively. Going beyond previous research, we showed that 40 Hz VS can evoke neuronal activity at this frequency in different phases of sleep while preserving sleep quality. Our method provides high experimental control for confounding factors commonly associated with gamma activity measurement. It opens up new possibilities to causally study high-frequent oscillatory brain activity and its associated functions during sleep, of particular relevance given the posited interplay between gamma activity, sleep, and memory. Importantly, it lays the groundwork for a potential improvement of VS as a clinical intervention against dementia.

Dementias such as Alzheimer’s disease (AD) are a major societal issue, which is expected to worsen as life expectancy and the percentage of elderly people in the global population increase. One approach to tackle disease progression that has recently attracted considerable attention is periodic sensory stimulation [1]. It shows promise in mitigating neuropathology and AD symptoms by entraining gamma-band (30–100 Hz) neuronal oscillations, which are related to memory and other cognitive functions [2]. In humans suffering from early AD, 40 Hz visual and/or auditory stimulation increased functional neuronal connectivity [3], improved disease biomarkers and cognition [4], and even reduced night-time active periods [5]. However, results regarding gamma activity and neurotoxic molecule clearance are mixed [3, 6], and authors suggested restricted stimulation duration as a limiting factor. Nonetheless, in most studies, patients already received stimulation for 1 hour a day over several weeks—a considerable portion of their daily lives. Thus, sensory stimulation can still be improved in terms of efficacy and usability.

A few pilot studies on healthy samples have proposed ways to make 40 Hz visual stimulation (VS) more effective, for example, by combining it with cognitive tasks [7] or modulating the light stimulus [8]. One avenue that has not yet been considered, which could increase stimulation duration while minimizing time attending to the therapy, is the application of 40 Hz VS during sleep. More importantly, this approach could take advantage of the activation of the glymphatic system to clear neurotoxic amyloid-beta molecules from the brain—a neural mechanism possibly shared by 40 Hz stimulation [9] and sleep [10, 11]. Beyond improved usability, a higher efficacy of 40 Hz VS in sleep is thus plausible as well.

In fact, this established link between the glymphatic system, dementia, and sleep [12] has already led to interesting advances in the field of Photobiomodulation therapy [13–15]. Photobiomodulation refers to the delivery of near-infrared light to selected brain regions through the scalp. It can be applied in human patients with AD to target a range of pathophysiological aspects, including the blood-brain barrier and glymphatic flow, and can be guided by electroencephalography (EEG) [16], The enhanced removal of amyloid-beta molecules is one of the main posited mechanisms of this therapeutic approach, which is why it pairs so well with sleep [14, 15]. First studies report that Photobiomodulation could enhance memory functions [17] and even improve sleep quality [18, 19] in individuals with cognitive decline. This application, while promising, is still in its infancy [20], and faces the inherent difficulty of light transmission through the thick scalp bone. In comparison, delivering red light through the much thinner closed eyelids would also stimulate the cortex, albeit via the sensory pathway. This is feasible, while also enabling 40 Hz oscillatory entrainment [21, 22], which may be additionally beneficial for glymphatic clearance [9]. Taken together, the abovementioned studies clearly encourage the exploration of red-light 40 Hz VS during sleep.

Such a paradigm is untested, though some related studies point toward potential feasibility. In sleep, two studies showed that periodic VS up to 10 Hz can entrain brain activity in the corresponding frequency [23, 24]. The magnitude of steady-state visually evoked potentials (SSVEPs) measured with EEG varied by sleep stage. At 40 Hz, auditory evoked potentials have been recorded in deep sleep stages [25, 26]. Finally, 40 Hz VS before sleep promoted sleep quality in insomnia patients [27, 28]. We can conclude that 40 Hz VS in sleep is worth investigating, but a feasibility test is warranted: It is unknown if VS can effectively modulate gamma activity during sleep, whether effects differ by sleep stage, and how the intervention may affect sleep quality. Moreover, measuring gamma-band EEG activity is particularly challenging due to confounds such as electrical, ocular, and muscular artifacts [29].

To address this knowledge gap, we conducted a proof-of-concept experiment. We expected that 40 Hz VS would evoke 40 Hz EEG oscillations through closed eyes in all stages including wakefulness (W), rapid-eye-movement (REM) sleep, and non-REM (NREM) stages 2 and 3, and those magnitudes would differ depending on the stage. For a comprehensive account of 40 Hz VS effects and to rule out confounding factors, we examined EEG signals in both the frequency and time domains. In addition, we explored the effects of 40 Hz VS on objective and subjective sleep quality. These objectives were addressed through an adequately powered within-subjects study, including one control and one experimental night of polysomnography (PSG).

## Methods

### Ethics

The study was approved by the ethical committee of the Technical University of Munich and complies with the Declaration of Helsinki. Written informed consent was obtained from all study participants, who were compensated with 100 € upon study completion.

### Design

**Figure 1.**
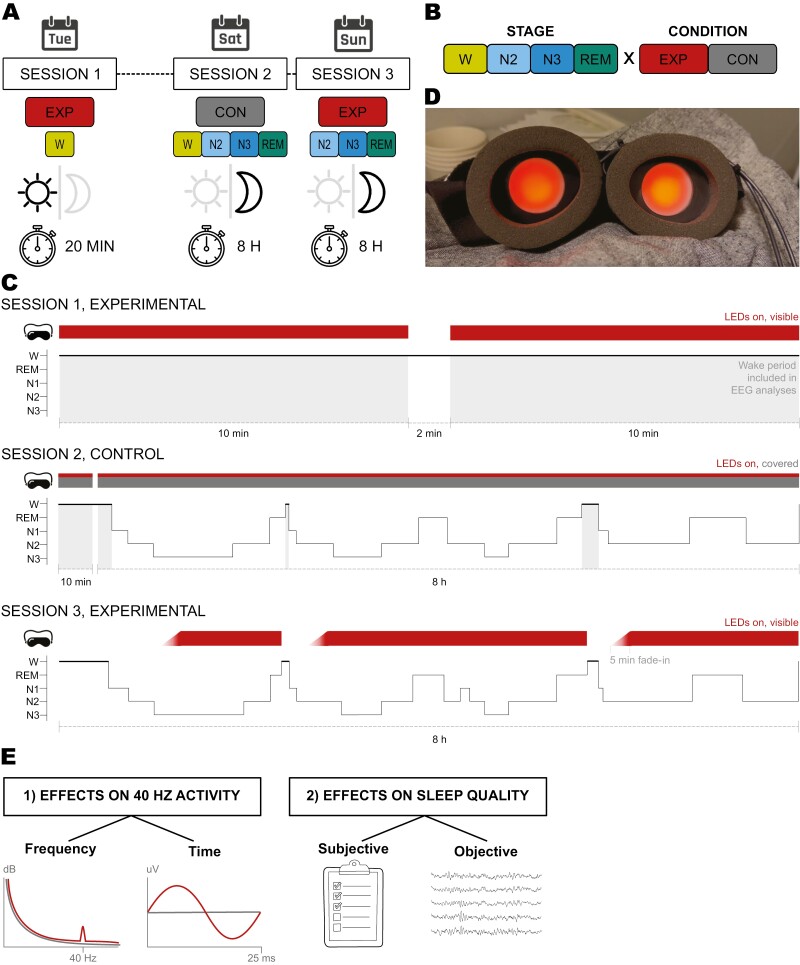
Study protocol and mask. (A) An overview of the characteristics of each experimental session, including day of the week (days only representative; weekday vs. weekend), condition, stages, day / night, and recording duration. (B) Study design. The variable stage has the categories awake (W), NREM 2 (N2), NREM 3 (N3), and REM. The variable condition has the categories control (CON) and experimental (EXP). (C) A representation of the three study sessions. Red bars = visible stimulation; red-grey bars = stimulation system ON, but no light visible to subjects; white-red gradients = stimulation fade-ins over 5 minutes; light-grey shaded areas = epochs marked as awake, stimulation system ON. Hypnograms are only exemplary. (D) Custom stimulation mask, consisting of a commercially available sleep mask with in-built LEDs (here, ON). (E) Schematic representation of the outcome variables. (1) Neuronal effects were analysed in the frequency and time domains; red curves represent expected effects in the experimental condition compared to control, in grey. (2) Effects of the stimulation on sleep were assessed via a questionnaire and Polysomnography.

The study followed a 2 × 4 within-subjects design with the factors condition (control [con], experimental [exp]) and stage (wakefulness [W], NREM 2 [N2], NREM 3 [N3], REM; Figure 1, A and B). In session 1, participants received VS during wakefulness only. In session 2, subjects underwent a control condition without visible stimulation; to reduce subject load and facilitate recruitment, this night also served as a baseline. VS during sleep was additionally applied in the second, experimental night (session 3). We collected data for the experimental W bin separately in session 1 instead of stimulating at the beginning or end of session 3, because directly before bedtime, light exposure could raise arousal levels and delay sleep onset, whereas in the morning, sleep inertia and higher PSG impedances could distort results. Experimenters were not blinded; the order of conditions was equal for all participants to mitigate the “First Night Effect” [30], and to spot any undiagnosed sleep disturbances via PSG before the intervention. Accordingly, sleep quality variables are reported as secondary.

### Sample

Exclusion criteria were assessed with a survey (Table 1) hosted on the online platform REDCap [31]. We used the Micro Munich Chronotype Questionnaire (μMCTQ [32]) to estimate chronotype, the Alcohol Use Disorders Identification Test (AUDIT [33]) for alcohol abuse, the Pittsburgh Sleep Quality Index (PSQI [34]) for average sleep quality, and Ishihara plates [35] at session 1 for color blindness. Simulation-based power analyses at standard levels of 1−β = 0.8 and α = 0.05 resulted in a sample of *N* = 30 to detect effects of at least *d* = 0.7 with pairwise comparison tests. Two participants dropped out of the study before reaching session 3 and were replaced with two new volunteers. The remaining sample of *N* = 30 comprised healthy young participants (age range: 20–30 years; M = 24.53 years, SD = 3.52 years); 19 females and 11 males, whose indicated gender matched their sex assigned at birth. One participant was left-handed, one was ambidextrous, and 28 were right-handed. On a scale from 0 (“Less than secondary education [no school degree or up to 7th grade]”) to 7 (“Doctorate or equivalent”), our sample was well-educated (range 3–6 points; M = 4.53, SD = 1.25). Baseline sleep quality was good (range 1–5 PSQI score; M = 3.7, SD = 1.09). Chronotypes, measured with the μMCTQ as the mid-sleep time on free days corrected for sleep debt (MSFsc), were intermediate on average (range 01:52 am–06:15 am, M = 04:19:30 am).

**Table 1.** Exclusion Criteria

Aspect	Screening modality	Exclusion criteria
Age	Self-report	<18 years or >35 years
Vision	Self-report, Ishihara test	Red-green colour blindness, any history of eye disease
Neurological disturbances	Self-report	Any history of neurological symptoms, especially epilepsy, migraine, stroke, brain tumour, concussion
Family history of epilepsy	Self-report	Any first-degree relative diagnosed with seizures
Sleep disturbances	Self-report, Pittsburgh Sleep Quality Index (PSQI)	Any symptoms in the past 6 months, especially insomnia, sleepwalking, bruxism, narcolepsy, restless legs syndrome, sleep apnoea; bad average sleep quality (PSQI > 5)
Psychiatric disturbances	Self-report	Any symptoms in the past 6 months, especially depressed mood, (hypo)mania, excessive worries, hallucinations or delusions, substance abuse, suicidal thoughts
Shift work	Self-report	Any shift work in the past month
Long-distance travel	Self-report	Any travel across 2 or more time zones in the past month
Substance use	Self-report, Alcohol Use Disorders Identification Test (AUDIT)	Any use of illicit drugs, cannabis, nicotine, or psychopharmacological medication in the past month; current alcohol abuse (AUDIT > 15)
Chronotype	Self-report, Munich Chronotype Questionnaire Micro (μMCTQ)	Chronotype measure MSFsc < 01:30 (“extremely early”) or > 06:30 (“extremely late”)

MSFsc, mid-sleep time on free days corrected for sleep debt.

### Procedure

The study procedure for each participant was as follows (Figure 1C). In session 1, at the target illuminance level, two VS blocks of 10 minutes were recorded while the participant remained awake with eyes closed. A short break between blocks helped maintain the participants’ alertness. Two nights were then scheduled at the laboratory on a subsequent weekend. In the week before, participants kept a constant sleep–wake schedule. Starting three days before the first night, participants refrained from unusual amounts of caffeine and any alcohol or drug intake. Participants arrived at the lab approximately one hour before their habitual bedtime on free days as indicated in the μMCTQ and adjusted if sleep habits had changed in the time between screening and session 2.

Upon arrival at the lab on session 2, participants performed a urine drug test (Drug-Screen Multi Test, nal von minden GmbH, Regensburg, Germany) and completed their usual sleep routine before PSG and the stimulation system was set up. Room lights were dimmed to a minimal level for preparation and turned off during the night. The mask was turned on upon recording start, its LEDs were covered with black tape. Thus, the setup was as similar as possible to session 3, except for the LED light not reaching the participants’ eyes—this enabled us to detect any electrical LED artifacts in the EEG. Participants remained seated with the mask on for ten minutes to record a wakefulness baseline, then laid down to sleep. They were given 8 hours of sleep opportunity before being awakened. If they woke up earlier naturally and could not fall back asleep, they could terminate the session then (this also applied to session 3). The mask was turned off upon recording termination, yielding the maximal number of trials for a solid baseline control condition. After setup removal, the Groningen Sleep Quality Scale was administered (GSQS [36]).

Participants returned in the evening of the same day or the day after for session 3. After nightly routines and experimental setup were completed, participants laid down to sleep right away. On this night, the intervention was delivered by a trained researcher actively monitoring PSG signals. At the first signs of N3 sleep, the researcher turned the VS on. To minimize arousal likelihood, illuminance levels gradually rose over 5 minutes following a cosine-windowed ramp pattern and were then kept constant overnight at the target level. In the case of arousal, VS was interrupted with a fade-out of 5 seconds and restarted when the participant fell back asleep: as soon as the researcher scored six consecutive 30-second PSG epochs as a stage other than W or N1, the VS was reinitiated with a fade-in. The delay until the first N3 phase and any interruptions inevitably led to fewer trials in the experimental condition compared to the control, but by leaving the stimulation on in the absence of arousals, as many trials as possible were recorded. In the morning, after eight hours of sleep opportunity and setup removal, the GSQS was administered.

### Intervention

A customized sleep mask with inbuilt LEDs externally linked to a microcontroller (Figure 1D) was used to deliver visual stimuli. The design of the system was derived from commercially available masks (e.g. https://noctura.com/) as well as previously published setups ([24]). The mask’s spongy surface in direct contact with the face (Figure 1D) allowed for an adjustment of the mask to the facial structure, and thus for sleeping in participants’ preferred positions. It featured high-wavelength LEDs perceived as red light: besides being most transmissible through the eyelids, red light is least disruptive to circadian rhythms [37]. The narrow spectral peak at 605 nm optimizes both eyelid transmission [38] and L-cone activation [39]. L-cones are the retinal cells most sensitive to high wavelengths, so a choice of wavelength close to L-cone peak sensitivity maximizes the amount of light processed by these cells and, consequently, by the visual system. The light was always temporally modulated at 40 Hz in a square-wave pattern and a 50% duty cycle. The average target illuminance was 80 lux at eye level, a value deemed appropriate by an independent pilot sample. Corneal irradiance could not be measured through closed eyes; based on previous models, the expected light levels are one log unit lower, resulting in approximately 8 lux [38].

For PSG and EEG recording and online sleep stage monitoring, we used the Neurofax PSG system, supported by Polaris.One software (Nihon Kohden Europe GmbH, Rosbach v.d.H., Germany). Gold cup electrodes were placed onto the mastoids (A1, A2), central EEG (C3, C4), chin EMG (LEMG, REMG), as well as diagonal EOG positions (LEOG, REOG), according to PSG standards. The EOG electrodes were placed approximately 3 cm away from the outer ocular canthi and covered with tape, so that the mask, closely covering participants’ eyes, would not interfere with signal capture. Occipito-parietal monopolar EEG channels (O1, Oz, O2, PO3, POz, and PO4) were used as the region of interest for primary statistical analyses. We performed offline sleep scoring automatedly using the Python library YASA, validated on over 30 000 hours of PSG across heterogeneous populations (V0.6.3 [40]).

### Outcomes

#### Primary outcome: 40 Hz power (dB).

The primary outcome was spectral density power at 40 Hz in dB, measured with EEG at the occipito-parietal region of interest and estimated by Fast-Fourier-Transform (FFT). It assesses how strongly the 40 Hz frequency in the EEG, corresponding to the VS frequency, contributes to the total signal. Lower values closer to zero reflect stronger power.

Following PSG standards, data were segmented into 30-second epochs and subjected to FFT. FFT outputs of all epochs were averaged and the value at 40 Hz was selected for statistical analyses (PSD40). EEG data preprocessing, conducted with MNE Python (V1.4.2 [41]), included the following steps: band-pass filtering (0.16–300 Hz); bad channel rejection; occipito-parietal electrode averaging; re-referencing to the mastoid average; bad trial rejection (>1 mV peak-to-peak amplitude OR <50% sleep scoring algorithm certainty OR stimulation duration <25 seconds); applying an FFT at epoch level using a Hamming window; averaging FFT outputs by condition and stage. For the baseline control condition, data from session 2 were used. For the experimental condition, data from session 3 were assigned to stage N2, N3, or REM bins; for the stage W bin, only data from session 1 were included (Figure 1A).

The signal-to-noise ratio (SNR) for PSD40 was computed for each condition and stage by dividing the power value at 40 Hz in dB by the surrounding values ([38 to 39.5 Hz] + [40.5 to 42 Hz]). This corrects for individual 1/f distributions in the EEG. An SNR value = <1 means that activity levels at 40 Hz and neighboring frequencies are similar; a higher value shows that 40 Hz activity is distinguishable from background activity.

#### Secondary outcomes: SSVEP amplitudes (μV).

We also conducted steady-state visually evoked potential (SSVEP) analyses of EEG data from the occipito-parietal region of interest in the time domain. Here, every 25 milliseconds segment time-locked to LEDs ON equals one trial; segments were averaged by condition and stage. Session 2 data were assigned to the baseline control condition, while sessions 1 and 3 were used for the experimental condition, as for PSD40. We chose SSVEP peak-to-trough amplitude in μV as an additional indicator of VS effect magnitude, with a higher amplitude reflecting a larger effect (SSVEPamp [42]). Because segments are time-locked to the flicker and of a duration matching the stimulation frequency, this variable directly represents the stimulus-evoked activity. Inspecting SSVEPs in the time domain allows us to average over a larger number of short segments, strengthening our claim that the EEG effects are in fact attributable to VS, not any high-frequency artifact such as muscle or ocular activity or other non-stationary influences. In experimental conditions, VS should elicit a sinusoidal SSVEP with a 25 milliseconds period (1 second divided by 40), while artifacts, which are not time-locked to the segments, should average out. Moreover, any electric artifacts from the LEDs would be made visible, since they would be time-locked to the segments, distorting the flat lines expected in control conditions (Supplementary Materials). In SSVEP analyses, no FFT was applied; segments with a signal range >100 μV peak-to-trough were rejected before averaging.

The SNR was computed as follows: for each condition and stage, take every 25 milliseconds segment and shuffle the order of data points; average segments to create a “random” SSVEP and calculate its peak-to-trough amplitude; repeat 100 times. The SNR is the ratio of the “true” SSVEP amplitude to the average of the “random” SSVEP amplitudes. An SNR value = <1 means that the averaged signal contains no clear temporal structure; a higher value shows that the signal is non-random.

#### Secondary outcomes: sleep quality.

Regarding secondary sleep variables, the Groningen Sleep Quality Questionnaire (GSQS) assesses the subjective quality of the previous night’s sleep, ranging from 0 (perfect sleep quality) to 14 (poor sleep quality). Standard objective parameters were computed with YASA[40]. Total Sleep Time (TST) quantifies the total duration of N1 + N2 + N3 + REM sleep in a night in minutes, ranging from 0 (a night spent awake) to 480 minutes (sleep duration equivalent to full sleep opportunity). Wake after sleep onset (W) quantifies the summed duration of wake periods overnight in minutes, within the span from the first to the last period of sleep. Its minimum is 0, i.e. the participant fully slept through; a higher value (max. 479 minutes) can point to a few longer wake periods or multiple short awakenings. Lastly, the duration of each sleep stage as a percentage of TST %(N1, . . . REM) is reported descriptively.

### Statistical analysis

For the main outcome PSD40, we defined five confirmatory hypotheses a priori: we postulated that PSD40 would be higher in the experimental condition compared to control in the four stages W, N2, N3, and REM (H1a—H4a) and that PSD40 in the experimental condition would differ between stages (H5a). SSVEPamp analyses followed analogous hypotheses (H1b–H5b). For secondary sleep quality analyses, we expected subjective and objective measures to differ between nights (H6–H8). The hypothesis was undirected since sleep quality in the second night could either be worse due to VS, or better due to the first-night effect. SNR values are reported descriptively as an additional source of signal quality information.

Statistical analyses were run in R (V4.3.0 [43]). For pairwise comparisons, if the test assumptions (normality, outliers) were met, we performed one-tailed (H1–H4) or two-tailed (H6-H8) pairwise *t*-tests; if not, we performed Wilcoxon signed-rank tests. For multilevel comparisons (H5), if assumptions (normality, outliers, sphericity) were met, we conducted a repeated-measures ANOVA; if not, we conducted a Friedman ANOVA. A significance threshold of α = 0.05 was selected for null hypothesis rejection. Tukey tests were employed for post-hoc pairwise contrasts, and Bonferroni-Holm corrected for multiple testing. The effect sizes—Cohen’s *d*_*z*_ for parametric tests, and rank biserial correlation *r* for non-parametric tests—were interpreted based on standard rules of thumb [44, 45] using the R package effectsize [46].

## Results

### 40 Hz VS increases 40 Hz EEG spectral power in W, N2, N3, and REM

**Figure 2.**
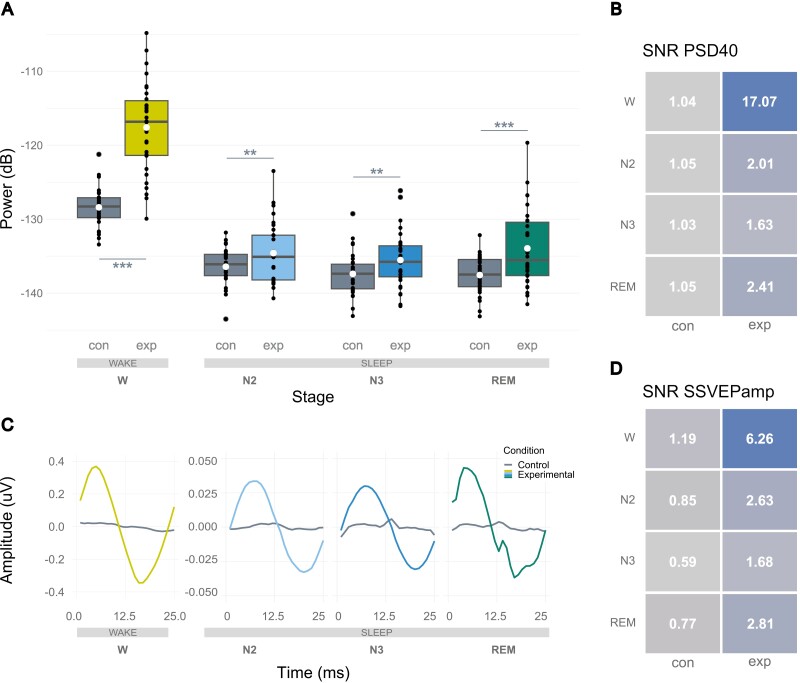
Primary results: Neuronal 40 Hz activity evoked in W, N2, N3, and REM. (A) Average spectral density power at 40 Hz (PSD40) across stages and conditions. White dots = means; grey lines = medians. ** = *p < *.01. *** = *p < *.001. (B) Signal-to-Noise Ratio values for the PSD40 variable, across stages and conditions. (C) Grand average Steady-State Visually Evoked Potentials across subjects, stages and conditions. Coloured lines = experimental conditions. (D) Signal-to-Noise Ratio values for the SSVEPamp variable, across stages and conditions.

Table 2 displays the descriptive statistics for spectral density power at 40 Hz (PSD40). Figure 2A shows the distributions of PSD40 per stage and condition. As expected, in the experimental condition compared to control, 40 Hz spectral power was increased in W (*p* < .001), N2 (*p* = .001), N3 (*p* = .002), and REM (*p* < .001). Effect sizes were large in W (*d*_*z*_ = 1.75; 95% CI [1.19; 2.31]) and REM (*r* = .94; 95% CI [0.88; 1]), and medium in N2 (*d*_*z*_ = 0.59; 95% CI [0.21; 0.97]) and N3 (*d*_*z*_ = 0.56; 95% CI [0.18; 0.94]). We can conclude that 40 Hz VS effectively increases spectral power in the equivalent frequency in wakefulness, deep NREM, and REM sleep.

**Table 2.** Descriptive Statistics for 40 Hz Spectral Power (PSD40)

Stage	Mean_con_	Mean_exp_	SD_con_	SD_exp_	Average # trials_con_	Average # trials_exp_
W	−128.40 dB	−117.60 dB	2.64 dB	6.17 dB	89.20	37.40
N2	−136.43 dB	−134.59 dB	2.51 dB	4.08 dB	403.27	218.83
N3	−137.39 dB	−135.53 dB	2.88 dB	3.78 dB	135.07	117.97
REM	−137.56 dB	−133.95 dB	2.54 dB	5.20 dB	157.47	118.30

^#^trials = average number of 30-second epochs factoring into analyses.

Additionally, in the experimental condition, PSD40 differed between stages as predicted (*p* < .001), with a large effect size (η^2^ = 0.9; 95% CI [0.87; 1]). However, *post-hoc* tests revealed that only stage W differed from all other stages, with no significant differences between the sleep stages N2, N3, and REM (*p*_*W-N2*_ < .001, *p*_*W-N3*_ < .001, *p*_*W-REM*_ < .001, all other *p* = 1). That is, 40 Hz power during stimulation was largest in W and similarly large between sleep stages.

The median SNR values for PSD40 are displayed in Figure 2B. Consistent with the aforementioned analyses, SNR values were close to one in the control condition and higher than one in the experimental condition, with a markedly higher value in W_exp_.

### 40 Hz VS evokes SSVEPs in W, N2, N3, and REM

Table 3 displays the descriptive statistics for Steady-State Visually Evoked Potential peak-to-trough amplitudes (SSVEPamp). Figure 2C shows the grand average SSVEPs per stage and condition. As predicted, in the experimental condition compared to control, SSVEP amplitudes were increased in W (*p* < .001), N2 (*p* < .001), N3 (*p* < .001), and REM (*p* < .001). Effect sizes were large in W (*r* = .99; 95% CI [0.97; 1]), N2 (*r* = .88; 95% CI [0.77; 1]), and REM (*r* = .82; 95% CI [0.66; 1]), and medium in N3 (*d*_*z*_ = 0.7; 95% CI [0.31; 1.09]). We can conclude that 40 Hz VS effects also translate into higher SSVEP amplitudes in wakefulness, deep NREM, and REM sleep.

**Table 3.** Descriptive Statistics for SSVEP Peak-to-Trough Amplitude (SSVEPamp)

Stage	Median_con_	Median_exp_	IQR_con_	IQR_exp_	Average # trials_con_	Average # trials_exp_
W	0.11 μV	0.96 μV	0.11 μV	0.95 μV	142847.3	47102.17
N2	0.02 μV	0.08 μV	0.02 μV	0.08 μV	493731.4	249354.27
N3	0.02 μV	0.07 μV	0.03 μV	0.06 μV	166411.4	142389.30
REM	0.02 μV	0.09 μV	0.03 μV	0.11 μV	197698.5	136729.37

^#^trials = average number of 25-millisecond segments factoring into analyses. IQR, interquartile range.

Additionally, in the experimental condition, SSVEPamp differed between stages (*p* < .001); data were in substantial agreement with the hypothesis (*w* = 0.68; 95% CI [0.62; 1]). However, post hoc tests revealed that only stage W differed from all other stages, with no significant difference between the sleep stages N2, N3, and REM (*p*_*W-N2*_ < .001, *p*_*W-N3*_ < .001, *p*_*W-REM*_ < .001, all other *p* = 1). That is, SSVEP amplitude during VS was largest in W and similarly large between sleep stages.

Median SNR values are displayed in Figure 2D. Consistent with the analyses above, SNR values were close to one in the control condition and higher than one in the experimental condition, with a markedly higher value in W_exp_.

### Sleep quality was comparable on both nights

**Figure 3.**
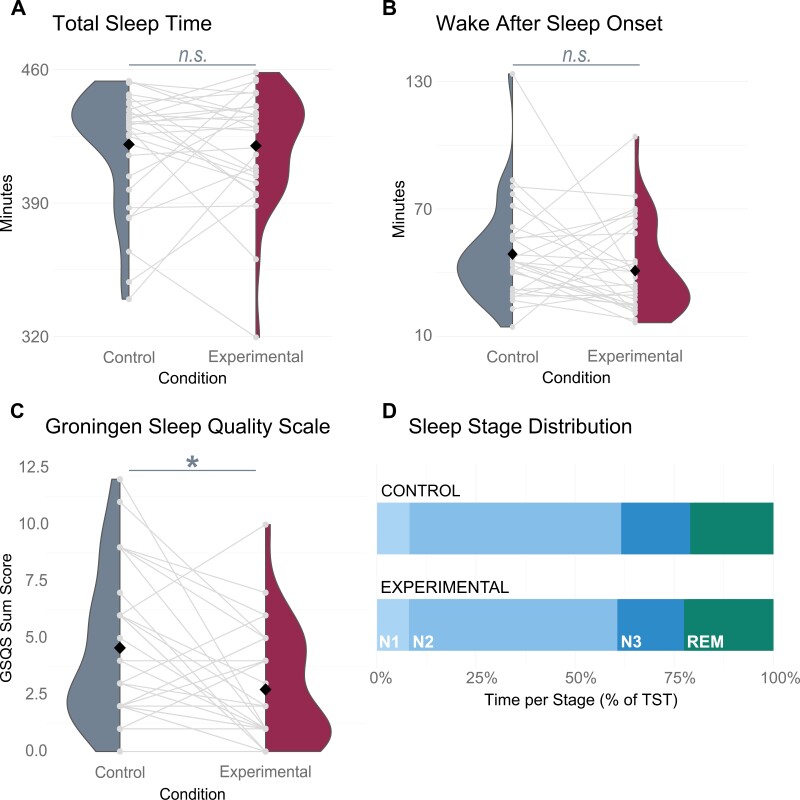
Secondary results: No difference between conditions in objective sleep parameters. (A) Distribution of TST in both nights. (B) Distribution of WASO in both nights. (C) Distribution of GSQS scores in both nights. Black diamonds = means; grey lines = individual subjects. (D) Distribution of sleep stages as a percentage of TST in both nights.

Figure 3 shows the distribution of sleep quality variables in the control and experimental conditions. As hypothesized, GSQS sum scores differed between conditions (Figure 3C; *p* = .010): on average, participants perceived sleep quality to be higher in the experimental (Mean_exp_ = 2.73, SD_exp_ = 2.56) than in the control night (Mean_con_ = 4.57, SD_con_ = 3.28). The effect was small (*d*_*z*_ = 0.48; 95% CI [0.11; 0.85]). Against expectations, TST did not differ between conditions (Figure 3A; *p* = .910), showing that on average, participants slept for a similarly long time in both the experimental (Mean_exp_ = 420.03 minutes, SD_exp_ = 30.12 minutes) and the control (Mean_con_ = 420.75 minutes, SD_con_ = 31.28 minutes) nights. WASO did not differ either (Figure 3B; *p* = .160), implying that on average, participants spent a similar amount of time awake overnight in both the experimental (Mean_exp_ = 40.95 minutes, SD_exp_ = 21.25 minutes) and the control (Mean_con_ = 48.75 minutes, SD_con_ = 23.34 minutes) nights. Lastly, Time per Stage descriptively pointed to a sleep stage distribution common in healthy, young sleepers (Figure 3D). Both nights were comparable, with lowest values for %N1 (Mean_con_ = 8.19%, Mean_exp_ = 8.05%), highest values for %N2 (Mean_con_ = 53.38%, Mean_exp_ = 52.55%), and both %N3 (Mean_con_ = 17.41%, Mean_exp_ = 16.82%) and %REM (Mean_con_ = 21.02%, Mean_exp_ = 22.58%) in-between.

## Discussion

In this within-participants proof-of-concept study, 30 healthy participants underwent periodic VS at 40 Hz through closed eyes while awake and while asleep. We found that stimulation successfully increased 40 Hz power in wakefulness, NREM, and REM sleep; time series analyses robust to artifacts corroborated these results. Sleep quality was comparable between the first, control night and the second, experimental night. This feasibility demonstration of 40 Hz VS in sleep lays the groundwork for a potential improvement of gamma sensory stimulation therapy.

### Going beyond previous work

The present study builds on prior work on periodic sensory stimulation in sleep by applying it in the gamma band, with a design suitable to exclude multiple confounding factors. Steady-state responses to 40 Hz stimuli have been recorded during sleep in the auditory domain: amplitudes were shown to decrease in NREM sleep, especially in the presence of slow-wave activity [25, 47]. This pattern is congruent with the lower SSVEP amplitudes we found in sleep. Sharon & Nir [24] reported that as opposed to slower (3/5 Hz) VS, SSVEPs in response to faster (8/10 Hz) VS were stronger in W than in NREM and REM sleep. In line with this pattern, the even faster 40 Hz VS we employed elicited stronger effects in W than in sleep. Sharon and Nir’s data was marked by lower TST, higher WASO, higher %N3, and lower %REM values than the present study’s, where values were closer to benchmarks [48]; this is likely attributable to their single-night experimental design. Norton et al. [23] only applied VS for a few minutes during the first two hours of sleep; SSVEP amplitudes in response to 7 Hz VS increased from W over light sleep to deep sleep. This pattern (wake > sleep) is opposite to ours (sleep < wake), which is not surprising, considering that Norton and colleagues only recorded SSVEPs during wakefulness for 2 minutes and mentioned eye adaptation as a confounding factor. Baseline theta activity is commonly stronger in NREM than W, as opposed to gamma, so the results can also be explained by the entrainment of endogenous brain activity as a possible mechanism driving EEG responses to periodic VS. Moreover, our findings replicate previous reports of 40 Hz activity enhancement by VS through closed eyes in wakefulness [21, 22], at a lower illuminance value that allows for sleep, while additionally ruling out confounding factors. In a pilot (*N* = 2), Jones et al. [21] reported that 40 Hz VS in wakefulness is less effective with eyes closed than eyes open, and argued that such a treatment should therefore be applied during wakefulness rather than sleep. Although we chose a lower illuminance to facilitate sleep, we succeeded at inducing 40 Hz gamma oscillations. Based on the medium to large effect sizes we found in different sleep stages and the rationale for gamma stimulation during sleep in clinical contexts, we claim that 40 Hz VS is well applicable during sleep.

### Methodological advances

Importantly, time series analyses revealed sinusoidal waveforms with a period of 25 milliseconds in experimental conditions (Figure 2C). These provide evidence for an effect resulting directly from 40 Hz VS instead of ocular or muscular artifacts, which are not time-locked to the visual flicker and therefore average to zero in the evoked response. Electrical artifacts from the mask LEDs could have been particularly problematic, as the on/off cycle of the LEDs is time-locked to the SSVEPs and would therefore not average out. Through our within-participants design with an electrically equivalent control condition, in which the mask was on but LEDs were covered, we could rule out this factor. Supplementary analyses using linear interpolation revealed that this processing step, which would eliminate any remaining artifacts, does not change the main results (Supplementary Materials). Our findings thereby open up the range of neuronal frequencies that can be reliably studied with VS during sleep, previously only explored up to 10 Hz [24]. Such slower oscillations have higher amplitudes and do not overlap with oculo-muscular frequencies; at faster frequencies, only a method with high experimental control as presented here can yield interpretable results.

Another advantage of this approach is the long stimulation duration achieved. At varying lengths (Figure S3), we were able to stimulate every participant in both deep NREM stages and REM. On average, 3.8 hours of stimulation were achieved overnight, nearly four times the duration common for patient studies while awake [3]. It is true that the increase in evoked 40 Hz oscillations and SNR was larger in wakefulness despite the lower stimulation duration; this is not surprising, considering the higher brain receptivity to external stimuli, attention, homogeneity of light exposure, and baseline gamma levels in this stage. Nonetheless, effect sizes during sleep were medium to large, and higher SNRs in the experimental condition showed that 40 Hz effects were discernible from background activity. This underpins the usability of this method for investigations on high-frequent neuronal oscillations during sleep. Independently of whether this method entrains endogenous oscillations or induces them, given the biological rationale for overnight interventions to target the glymphatic system and the possibility that intervention length is a key factor driving clinical effects [4, 6], these findings also uncover a potential for improving gamma sensory stimulation in clinical settings.

Moreover, our data provide a preliminary indicator for the feasibility of this method in terms of sleep quality. As can be expected in a laboratory setting, participants spent slightly more time in W and N1 overnight compared to benchmark values for healthy people [48], but this was true for both nights equally, and the overall sleep architecture was preserved (Figure 3D). We had expected both nights to differ regarding a priori selected parameters of objective sleep quality, but they did not. Post-hoc exploratory inspection of other sleep parameters revealed that REM sleep latency was shorter on the experimental night compared to the control (*p* = .01) and that descriptively, rapid-eye movement, spindle, and slow oscillation parameters were comparable across both nights (Figure S4). The First-Night Effect hypothesis would postulate better sleep quality on the second night, which was only the case at a subjective level; perhaps the VS itself elicited a sort of first-night effect at an objective level. Although these results must be interpreted with caution, considering the non-randomized design and possible feelings of social expectation, we at least observed that the intervention administered on the second night affected objective and subjective sleep quality equally to, or less than, the First-Night Effect in night one. Since demonstrating feasibility was our main goal, these findings encourage further research using this method.

### Limitations and outlook

One clear limitation of this study is the lack of an adaptation night, randomization, and blinding, precluding a fair comparison between conditions regarding sleep quality. Further research building on this proof-of-concept could aim to reduce any remaining intervention impacts on sleep and examine whether participants can fully habituate over multiple sessions. Although the use of an automated sleep scoring algorithm may be seen as a limitation, compared to the “gold standard” of manual scoring, it makes our results reproducible. Such an algorithm may be useful in a follow-up study to automatize stimulation onset. Moreover, future studies could add a camera to the mask to control for eye openings, and systematically inquire about lucid dreams, which have previously been reported in a study employing 40 Hz transcranial electrical stimulation in REM sleep [49] (anecdotally, in our study, no participant reported such an occurrence). Furthermore, while we chose a standard PSG setup to facilitate sleep, future studies may employ high-density EEG to investigate how SSVEPs spread across cortical regions. Lastly, the present design was limited to one frequency and one illuminance value, not accounting for individual differences in eyelid transmission and light sensitivity. With feasibility demonstrated, future work can systematically explore the parameter space and inter-individual variability. Particularly, parameters should be adapted to elderly participants, as age-related lens yellowing and altered baseline gamma levels can increase the need for personalization.

## Conclusion

To conclude, in our proof-of-concept study, delivering 40 Hz VS during sleep was feasible. The modulation of high-frequent brain oscillations in sleep using only light, the stimulation duration thereby achieved, and the methodological exclusion of confounding variables constitute this study’s main contributions to the field. The present findings enable us to take visual neuromodulation a step further into the realm of sleep: manipulating gamma oscillations has already proven useful to uncover neuronal mechanisms of memory during wakefulness [50], but considering the interplay between gamma oscillations, memory, and sleep [51], stimulating at gamma frequencies during sleep could generate important evidence. These functions are particularly relevant when impaired, as is the case in dementia. On a translational level, given the posited mechanism of neurotoxic molecule clearance shared by sleep and 40 Hz stimulation [9, 11], and the apparent need for a long stimulation duration [6], the method presented here could potentially increase the therapeutic value of gamma sensory stimulation for patients in early stages of dementia, including AD. Leading researchers call for the development of non-pharmaceutical approaches to complement current therapies in a scalable and equitable manner [52]; our findings encourage further research to test if gamma sensory stimulation in sleep could one day fit this vision.

## Supplementary material

Supplementary material is available at *SLEEP* online.

zsae299_suppl_Supplementary_Material

## Data Availability

A repository at Open Science Framework contains the preregistration and data (https://osf.io/gq73d/). Study materials and code are available on GitHub (https://github.com/tscnlab/Gamma-Sleep).
