# Supplementary material for: 40 Hz visual stimulation during sleep evokes neuronal gamma activity in NREM and REM stages
Source: Sleep. 2024 Dec 19;48(3):zsae299. doi: 10.1093/sleep/zsae299 (PMC11893540; doi:10.1093/sleep/zsae299)
Supplement: zsae299_suppl_Supplementary_Material [file zsae299_suppl_supplementary_material.docx]

*SUPPLEMENTARY MATERIAL*

40 Hz Visual Stimulation During Sleep Evokes Neuronal Gamma Activity in NREM and REM Stages

**Authors**

Laura Hainke ^1,2,3^

James Dowsett ^9^

Manuel Spitschan ^3,4,5^ **^*^**

Josef Priller ^1,6,7,8^ **^*^**

** Equal contribution*

**Affiliations**

^1^ Department of Psychiatry and Psychotherapy, TUM School of Medicine and Health, Technical University of Munich, Munich, Germany

^2^ Department of Psychology, Ludwig Maximilian University, Munich, Germany

^3^ Department of Health and Sport Sciences, TUM School of Medicine and Health, Technical University of Munich, Munich, Germany

^4^ Translational Sensory & Circadian Neuroscience, Max Planck Institute for Biological Cybernetics, Tübingen, Germany

^5^ TUM Institute of Advanced Study (TUM-IAS), Technical University of Munich, Garching, Germany

^6^ Neuropsychiatry, Charité – Universitätsmedizin Berlin and DZNE, Berlin, Germany

^7^ University of Edinburgh and UK DRI, Edinburgh, UK

^8^ German Center for Mental Health (DZPG), Munich, Germany

^9^ Department of Psychology, University of Stirling, Stirling, Scotland, UK

**Addresses**

LH & JP (work was conducted here):
Klinikum rechts der Isar

Klinik und Poliklinik für Psychiatrie und Psychotherapie

Ismaninger Str. 22

81675 Munich, Germany

JD (james.dowsett@stir.ac.uk):

Psychology Department

Faculty of Natural Sciences

University of Stirling

Stirling, FK9 4LA, Scotland UK

MS:

Technical University of Munich

TUM School of Medicine and Health

Department Health and Sport Sciences

Assistant Professorship of Chronobiology & Health
Georg-Brauchle-Ring 60/62

80992 Munich, Germany

**Corresponding authors**

Laura Hainke (laura.hainke@tum.de), Manuel Spitschan (manuel.spitschan@tum.de) or Josef Priller (josef.priller@tum.de)

[**Rationale for linear interpolation**](#_zctqz7zdlpm) 1

[**Results**](#_6nglovgbke1z) 1

[**Tables**](#_txotkfx5frvx) 1

[Table S1 Outcomes of confirmatory analyses with and without linear interpolation.](#_bra0gbkih8jp) 2

[Table S2 Signal-to-Noise Ratios with and without linear interpolation.](#_svxn7xqwfzle) 2

[**Figures**](#_gpu3lkqc2czy) 3

[Figure S1 Example SSVEP affected by artifacts.](#_7v6q89bsbjnu) 3

[Figure S2 Average SSVEPs after linear interpolation.](#_8m112wu87fp5) 3

[Figure S3 Stimulation duration per stage, experimental condition.](#_ber44rxqd58y) 4

# **Rationale for linear interpolation**

Linear interpolation refers to a data cleaning step employed to remove periodic artifacts. Here, such artifacts have occasionally been caused by the LEDs of the custom sleep mask placed directly over subjects’ eyes, and therefore close to EEG electrodes. At the intended stimulation frequency of 40 Hz and a duty cycle of 50 %, the LEDs were programmed to turn ON at timepoint 0 of each 25 ms segment, and OFF at 12.5 ms. The mask was built to be shielded as well as possible, however, SSVEPs were computed from a very high number of segments. While this is favourable to averaging out noise overall, any electric contamination by the LEDs would be very consistent in timing, and therefore visible in the averaged time series.

Figure S1 shows an example SSVEP exceptionally affected by this issue, with one downward and one upward peak at the timepoints of LED ON/OFF distorting the underlying sinusoidal waveform. The red dashed lines symbolise the linear interpolation procedure: Essentially, datapoints at the beginning (timepoints [-1;3]) and middle (timepoints [11;15]) of each segment are replaced with a straight line. Averaging across “cleaned” segments results in a sinusoidal form, without residuals of LED ON/OFF artifacts.

This procedure was not defined in the preregistration, since pilot data were not affected by artifacts. In fact, neither was the majority of the experimental datasets. Given that the presence or absence of artifacts was constant within participants, the control condition was electrically equivalent, and comparisons between conditions were carried out within-subjects, artifacts did not distort pairwise comparison results. For those reasons, linear interpolation was not applied to main analyses. Nevertheless, since some SSVEPs showed these characteristic peaks, we report results with this processing step applied here.

# **Results**

The interpretation of confirmatory analyses for PSD40 and Signal-to-Noise Ratios is not changed after applying linear interpolation (Table S1, Table S2). Figure S2 shows the grand average SSVEPs across stages and conditions after linear interpolation. Compared to the equivalent SSVEPs without interpolation (Figure 2C), curves are slightly smoother but retain their overall shape.

# **Tables**

|  | **No Interpolation** | | | **With Interpolation** | | |
| --- | --- | --- | --- | --- | --- | --- |
| **Hypothesis** | **Outcome** | **P-value** | **Effect size** | **Outcome** | **P-value** | **Effect size** |
| H1a | Accepted | <0.001 | *d_z_* = 1.75 | Accepted | <0.001 | *d_z_* = 1.61 |
| H2a | Accepted | 0.001 | *d_z_* = 0.59 | Accepted | 0.001 | *d_z_* = 0.59 |
| H3a | Accepted | 0.002 | *d_z_* = 0.56 | Accepted | 0.001 | *d_z_* = 0.59 |
| H4a | Accepted | <0.001 | *r* = 0.94 | Accepted | <0.001 | *r* = 0.96 |
| H5a | Accepted | <0.001 | 𝜂^2^ = 0.9 | Accepted | <0.001 | 𝜂^2^ = 0.9 |

## **Table S1 Outcomes of confirmatory analyses with and without linear interpolation.**

Outcome = hypothesis accepted or rejected at a significance threshold of *p* < .05. Effect sizes: Cohen’s d_z_ for paired-samples t-tests, rank biserial correlation *r* for Wilcoxon signed rank tests, eta squared 𝜂^2^ for repeated-measures ANOVA.

|  | **No Interpolation** | | | | **With Interpolation** | | | |
| --- | --- | --- | --- | --- | --- | --- | --- | --- |
|  | **PSD40** | | **SSVEPamp** | | **PSD40** | | **SSVEPamp** | |
| **Stage** | **SNR_con_** | **SNR_exp_** | **SNR_con_** | **SNR_exp_** | **SNR_con_** | **SNR_exp_** | **SNR_con_** | **SNR_exp_** |
| W | 1.04 | 17.07 | 1.19 | 6.26 | 1.03 | 16.04 | 1.26 | 6.52 |
| N2 | 1.05 | 2.01 | 0.85 | 2.63 | 1.05 | 1.99 | 0.74 | 2.26 |
| N3 | 1.03 | 1.63 | 0.59 | 1.68 | 1.04 | 1.59 | 0.50 | 1.45 |
| REM | 1.05 | 2.41 | 0.77 | 2.81 | 1.07 | 2.28 | 0.53 | 1.99 |

## **Table S2 Signal-to-Noise Ratios with and without linear interpolation.**

SNR = Signal-to-Noise Ratio; PSD40 = 40 Hz spectral power; SSVEPamp = Steady-State Visually Evoked Potential peak-to-trough amplitude; con = control condition; exp = experimental condition; W = wakefulness; N2 = NREM sleep stage 2; N3 = NREM sleep stage 3; REM = Rapid Eye Movement sleep stage.

# **
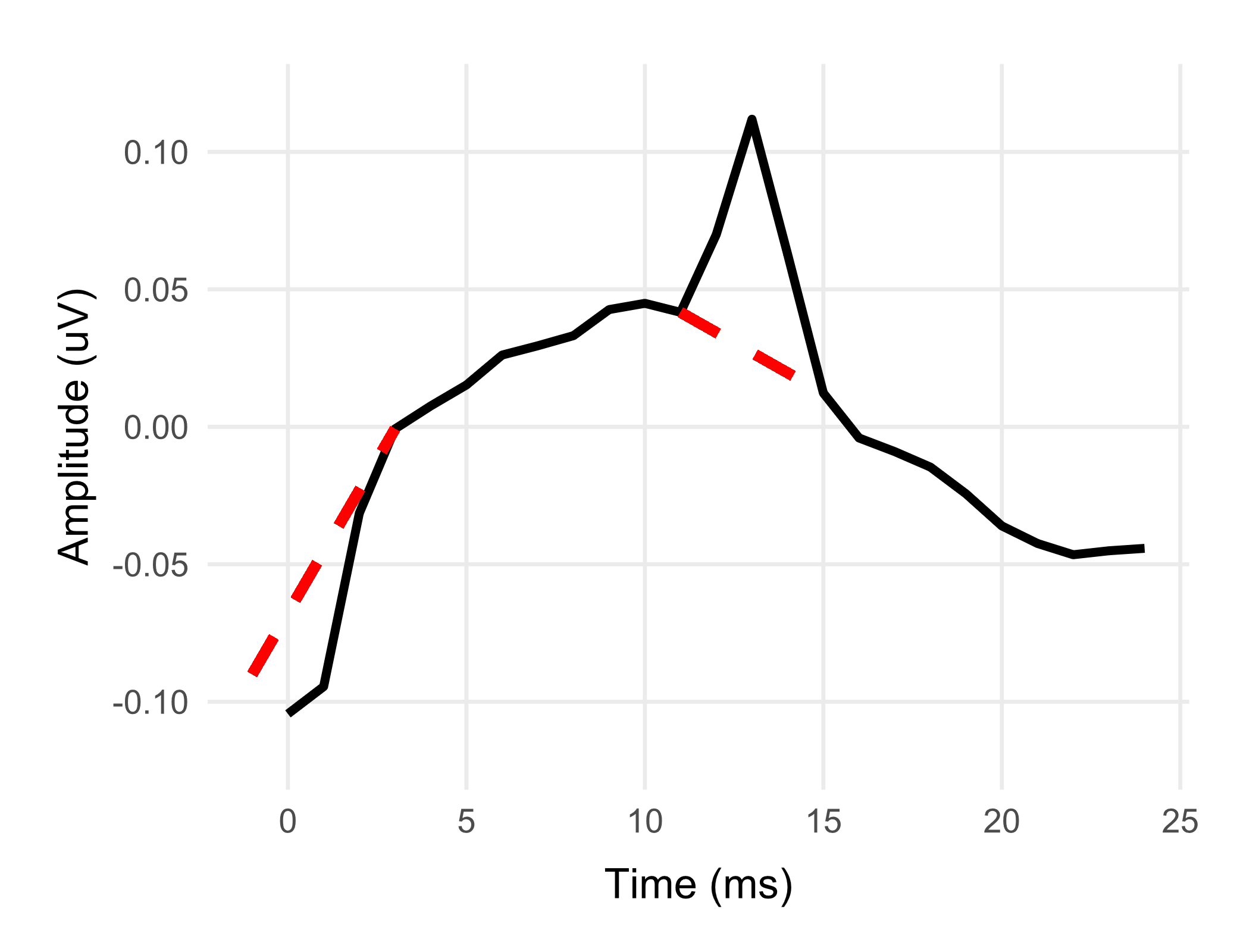
Figures**

## **Figure S1 Example SSVEP affected by artifacts.**

A sinusoidal SSVEP is distorted by one peak at the beginning and one in the middle of the time series. Red dashed lines represent the replacement of data with a straight line.

*Alt text:* With time in milliseconds on the x-axis and amplitude in microvolt on the y axis, an example SSVEP curve is shown in black, and the data cleaning step in red.


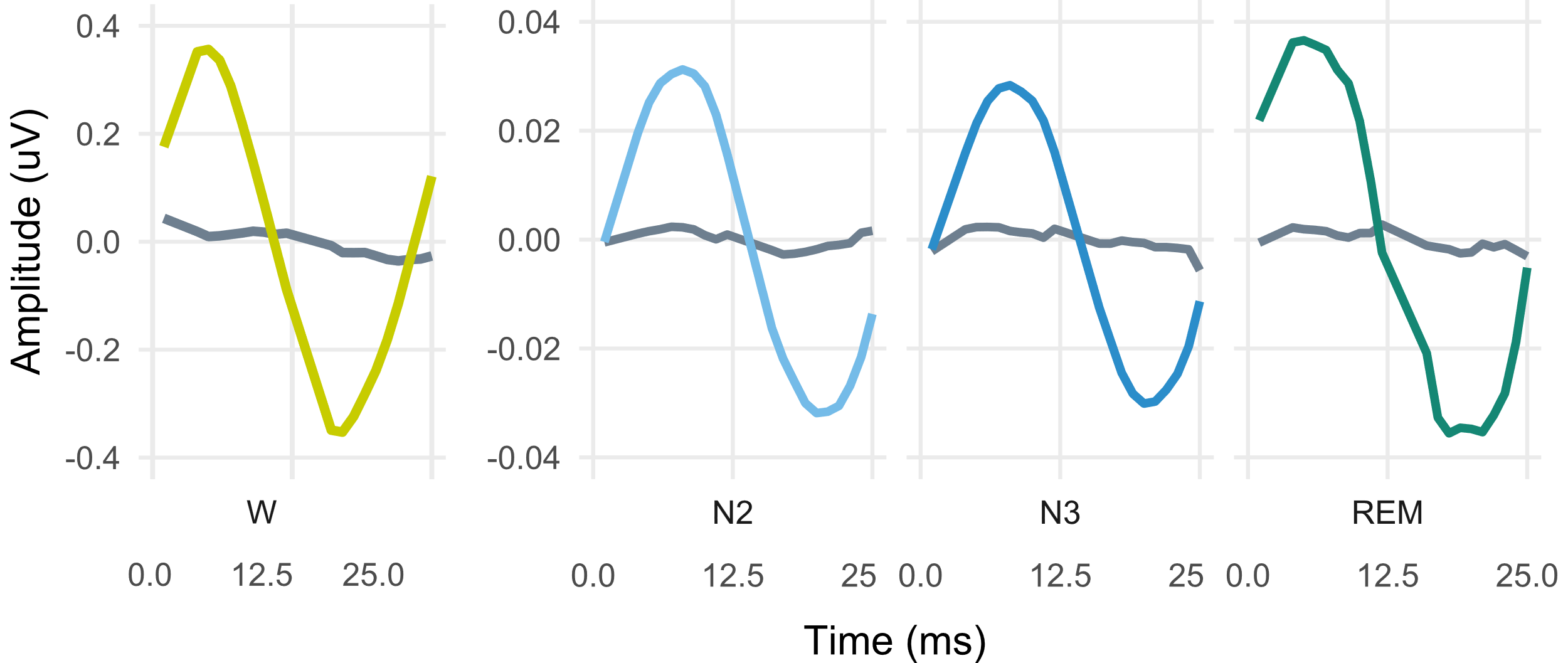


**Figure S2 Average SSVEPs after linear interpolation.**

SSVEPs averaged across subjects, per stage and condition. Coloured lines indicate experimental conditions, grey lines indicate control conditions.

*Alt text:* Experimental condition SSVEPs show a sinusoidal form with higher amplitude compared to flat lines in the control condition. They look very similar to the SSVEPs without this processing step applied.


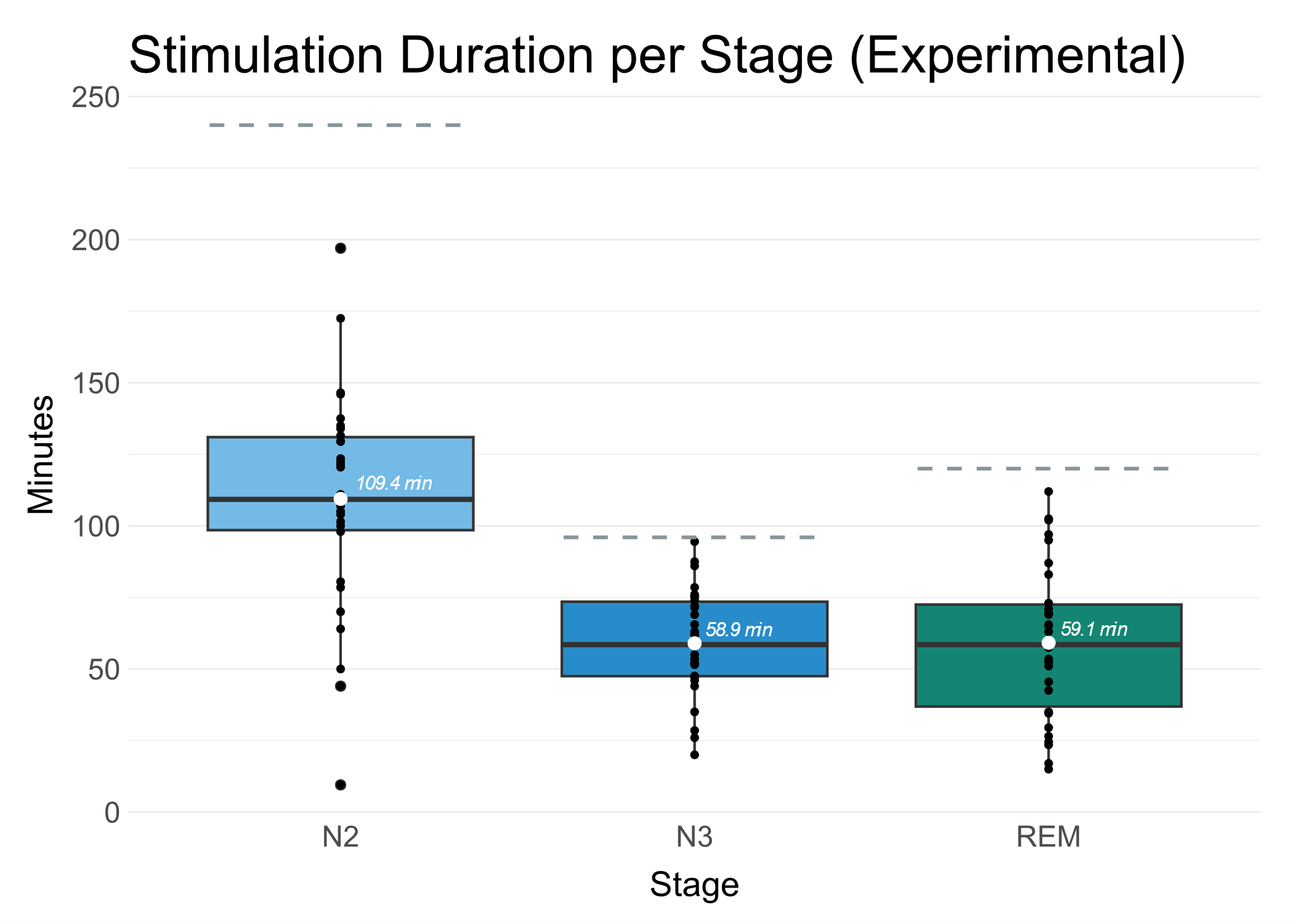


## **Figure S3 Stimulation duration per stage, experimental condition.**

Distribution of stimulated time per sleep stage across subjects, in the experimental condition. Dotted lines represent the usual time spent per night in a given stage. White dots mark mean values.

*Alt text:* Three boxplots, for N2, N3, and REM, respectively.

**
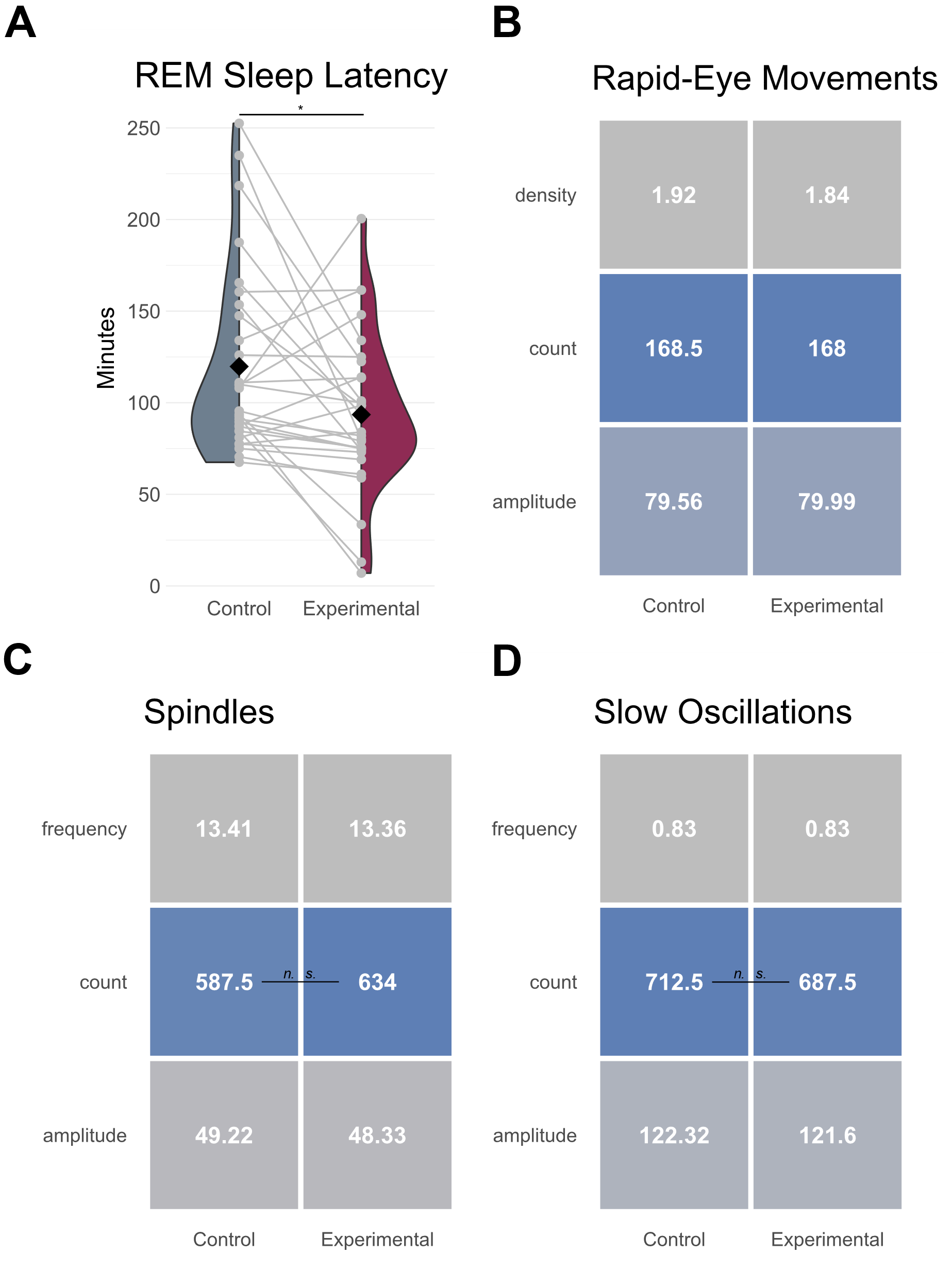
**

## **Figure S4 Supplementary sleep parameters.**

Post-hoc inspected sleep parameters, all calculated with the YASA package using default parameters. **A**: Distribution of REM sleep stage latency in both nights. A post-hoc paired t-test revealed that values were on average significantly (*) lower in the experimental night (*p* **=** .01). Black diamonds = mean values. **B**: Median values of rapid-eye movement parameters in the REM stage. Density = eye movements per minute; count = absolute number of detected eye movements; amplitude = peak-to-peak amplitude of eye movements in microvolt. **C**: Median values of sleep spindle parameters aggregated over N2 and N3 stages. Frequency = average oscillation frequency in Hz; count = absolute number of detected spindles; amplitude = average peak-to-peak amplitude of spindles in microvolt. A *post-hoc* paired t-test clarified that the spindle count did not significantly differ between conditions (*n.s.*). **D**: Median values of slow oscillation parameters aggregated over N2 and N3 stages. Frequency = average oscillation frequency in Hz; count = absolute number of detected slow oscillations; amplitude = average peak-to-peak amplitude of slow oscillations in microvolt. A post-hoc paired t-test clarified that the slow oscillation count did not significantly differ between conditions (*n.s.*).

*Alt text:* One half-violin plot for REM sleep latency; coloured tile plots for rapid eye movements, spindles, and slow oscillations parameters, with grey values indicating lower values and blue indicating higher values.
